# A comparison of fragmenting lead-based and lead-free bullets for aerial shooting of wild pigs

**DOI:** 10.1371/journal.pone.0247785

**Published:** 2021-03-11

**Authors:** Jordan O. Hampton, Grant Eccles, Rob Hunt, Andrew J. Bengsen, Andrew L. Perry, Steve Parker, Corissa J. Miller, Steve K. Joslyn, Sigbjørn Stokke, Jon M. Arnemo, Quentin Hart

**Affiliations:** 1 Faculty of Veterinary and Agricultural Sciences, University of Melbourne, Parkville, Australia; 2 School of Veterinary and Life Sciences, Murdoch University, Murdoch, Western Australia, Australia; 3 New South Wales National Parks and Wildlife Service, Hurstville, New South Wales, Australia; 4 Vertebrate Pest Research Unit, NSW Department of Primary Industries, Orange, NSW, Australia; 5 VetDB, Perth, Western Australia, Australia; 6 Norwegian Institute for Nature Research, Trondheim, Norway; 7 Department of Forestry and Wildlife Management, Faculty of Applied Ecology and Agricultural Sciences, Inland Norway University of Applied Sciences, Koppang, Norway; 8 Department of Wildlife, Fish and Environmental Studies, Swedish University of Agricultural Sciences, Umeå, Sweden; 9 New South Wales Department of Primary Industries, Queanbeyan, New South Wales, Australia; University of Nicolaus Copernicus in Torun, POLAND

## Abstract

In response to the health threats posed by toxic lead to humans, scavenging wildlife and the environment, there is currently a focus on transitioning from lead-based to lead-free bullets for shooting of wild animals. We compared efficiency metrics and terminal ballistic performance for lead-based and lead-free (non-lead) bullets for aerial shooting of wild pigs (*Sus scrofa*) in eastern Australia. Ballistic testing revealed that lead-based and lead-free bullets achieved similar performance in precision and muzzle kinetic energy (E_0_) levels (3337.2 J and 3345.7 J, respectively). An aerial shooting trial was conducted with wild pigs shot with one type of lead-based and one type of lead-free bullets under identical conditions. Observations were made from 859 shooting events (*n* = 430 and 429 respectively), with a sub-set of pigs examined via gross post-mortem (*n* = 100 and 108 respectively), and a further sub-set examined via radiography (*n* = 94 and 101 respectively). The mean number of bullets fired per pig killed did not differ greatly between lead-based and lead-free bullets respectively (4.09 vs 3.91), nor did the mean number of bullet wound tracts in each animal via post-mortem inspection (3.29 vs 2.98). However, radiography revealed a higher average number of fragments per animal (median >300 vs median = 55) and a broader distribution of fragments with lead-based bullets. Our results suggest that lead-based and lead-free bullets are similarly effective for aerial shooting of wild pigs, but that the bullet types behave differently, with lead-based bullets displaying a higher degree of fragmentation. These results suggest that aerial shooting may be a particularly important contributor to scavenging wildlife being exposed to lead and that investigation of lead-free bullets for this use should continue.

## Introduction

In response to the health threats posed by toxic lead to humans and scavenging wildlife, there is currently a focus on transitioning from lead-based to lead-free (non-lead) bullets for shooting (i.e., harvesting, culling, recreational hunting) of wild animals [1, 2]. Concurrently, attention devoted to animal welfare in wildlife management has increased markedly, including for shooting methods [3, 4]. As a result, there has been considerable scrutiny of animal welfare implications of a transition to lead-free products. Concerns that newly developed bullet technology may not achieve existing animal welfare standards have impeded the adoption of lead-free bullets [5]. Consequently, there is a requirement for evidence-based assessment of animal welfare impacts for new lead-free bullet technology [6].

Aerial (helicopter) shooting (aerial gunning) is a professional wildlife control method used to kill millions of wild animals of at least 23 species worldwide every year [7]. Aerial shooting typically involves large-bodied invasive mammal species being shot multiple times with semi-automatic rifles [8] or shotguns [9]. Aerial shooting is used particularly extensively in Australia, where it is the preferred population management tool over much of the continent for several species of introduced ungulates [10], including *Sus scrofa*, known as wild pigs, feral pigs, feral swine, wild boars or wild hogs [11–13]. An important distinction between culling (including aerial shooting) and consumptive shooting practices (hunting and harvesting) is that the former results in carcasses being ‘left to lie’ (‘culling-to-waste’) [14]. Lead exposure of scavengers from bullet fragments in carcasses left to lie is likely to be a widespread but under-recognized global problem [14–16].

Wild pigs have become a growing focus for wildlife management in many parts of the world. They are considered a pest species across much of their range globally and cause damage to natural and anthropogenic ecosystems [17]. Their impacts have been documented in Australia [18], North America [19], South America [20], and on many smaller islands. Aside from traditional environmental and agricultural damage, wild pigs are also seen as a threat due to their transmission of infectious diseases, such as African swine fever [21]. This species is commonly targeted for culling where they are invasive, covering a wide global distribution [22]. In their native range, including mainland Europe, wild pigs are managed as a hunting resource [23, 24]. Hence, whether for recreational, commercial or professional reasons, many wild pigs are shot worldwide every year. Aerial shooting is widely used for wild pig control in Australia [11, 12], the US [19, 25], New Zealand [26], and many offshore islands [27]. Conservation estate inhabited by wild pigs that is often targeted for aerial shooting include sensitive wetlands such as RAMSAR sites (wetlands of international importance) [28] where obligations exist for land managers to minimize the presence of pollutants such as lead and other heavy metals [29].

To our knowledge, no published studies have assessed the efficacy of lead-free bullets for aerial shooting or the lead exposure risks for scavenging wildlife associated with using lead-based bullets for this purpose. However, risks to scavenging raptors have been discussed from the use of other aerial shooting methods, e.g. use of lead shot for the control of coyotes (*Canis latrans*) in the western United States [30]. Here, we provide the first report comparing lead-based and lead-free ammunition for aerial shooting, using wild pigs in eastern Australia. The aim of the study was to assess the efficacy and terminal ballistic characteristics of one lead-based and one lead-free bullet type in .308 Winchester^®^ cartridges under conditions typical of aerial shooting in Australia. We did not aim to test lead-based and lead-free bullets in general.

## Materials and methods

The present study comprised two stages: (i) ballistic testing using inanimate targets, and (ii) a study of aerial shooting outcomes.

### Ballistic testing

Our ballistics trials were conducted on a public shooting range near Nowra, NSW, eastern Australia, in October 2018. Ballistic tests were performed on a recently developed non-lead bullet of appropriate size and design for wild pigs and the results were compared to lead-based bullets traditionally used and approved by the Feral Animal Aerial Shooting Team (FAAST) of the NSW National Parks and Wildlife Service [31] for aerial shooting of wild pigs. We used two types of factory-loaded ammunition in .308 Winchester^®^ cartridges: 1) lead-based Speer^®^ 130 gr non-bonded core hollow-point bullets (Speer Ammunition, Lewiston, Idaho, US), and 2) lead-free copper-based Controlled Chaos^®^ 115 grain hollow-point ammunition (Lehigh Defense, Quakertown, Pennsylvania, US). Lead-based bullets were of a frangible (‘varmint’) lead-core design typically associated with extensive fragmentation [32]. Lead-free bullets were of a fragmenting design not previously described in published literature, but intended to break into many fragments to minimize pass-through energy [33]. The design of the lead-free bullets assessed was considerably different to homogenous monolithic copper bullets described in many studies [34, 35]. We performed ballistic testing using paper bullseye targets and a chronograph to assess precision and muzzle kinetic energy.

**Fig 1:**
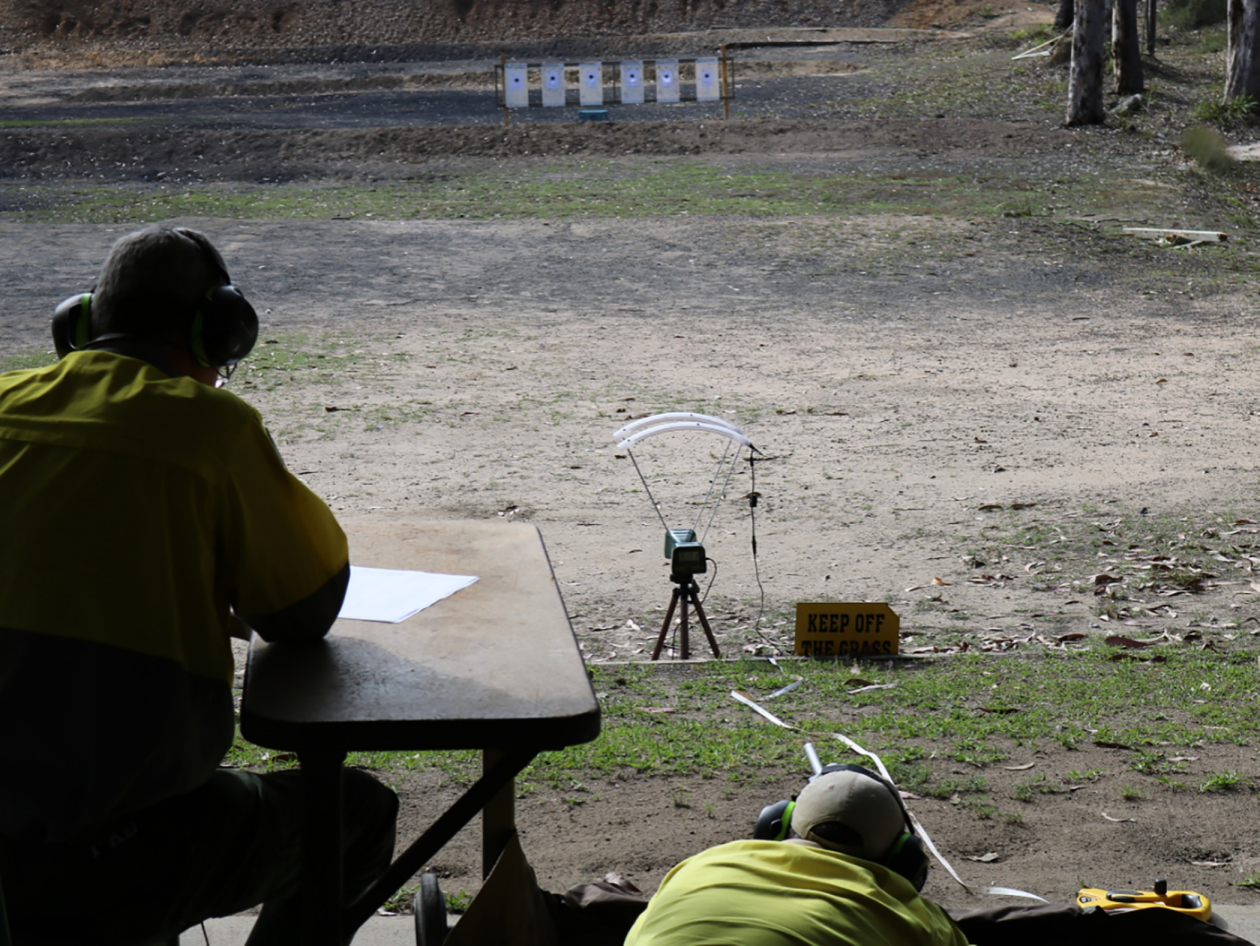
Shooting range testing of lead-based and lead-free bullets. Photographs of the use of a standardized shooting approach using paper targets to assess accuracy and precision and a chronograph to measure muzzle velocity.

Two five-shot groups were measured to assess precision, with group size calculated according to [6, 36]. A Caldwell Ballistic Precision^®^ chronograph (Caldwell Shooting Supplies, Colombia, Missouri, US) (Fig 1) was used to measure bullet velocity at the level of the rifle muzzle and allow calculation of kinetic energy (E_K_) at the level of the muzzle (E_0_) [37] for 10 shots from each bullet type.

### Aerial shooting

Our aerial shooting trial was conducted on conservation estate (Gwydir Wetlands State Conservation Area) near Moree, New South Wales, eastern Australia (29°18’56S, 149°18’38E) in May 2020. The reserve had a semi-tropical climate; vegetation was characteristic of diverse floodplain wetland communities in the Murray-Darling Basin drainage division [38]. The site had historically high wild pig densities and had been regularly targeted for aerial shooting programs [39].

Helicopter shooting operations targeted all observed wild pigs, adhering to the current Australian national model standard operating procedure [40] and Civil Aviation Safety Authority regulations. The shooting of large mammals from helicopters in Australia is governed by similar procedures regardless of species [40, 41]. Shooting procedures were, therefore, almost identical to those described in past studies [7, 8]. Briefly, shooters operated from an Airbus H125^®^ helicopter (Airbus Helicopters, Marignane, France). Two shooters were used, and both were professional aerial marksmen accredited under the FAAST program [31]. All shooting occurred during the hours of daylight (0800–1700). Shooters used a FN^®^ SCAR-H LB semi-automatic rifle (FN, McLean, VA, USA) chambered in .308 Winchester^®^ with an Aimpoint^®^ CompM4 red-dot sight without magnification (Aimpoint, Malmö, Sweden), zeroed at 30 m.

We were unable to collect ante-mortem (before death) data from wild pigs that were shot. This was due to inherent difficulties associated with examining animals soon after shooting when using an aerial platform [7, 8] and procedural protocols precluding having an observer in the shooting helicopter or the use of worn cameras to collect ante-mortem data [42]. This also precluded quantifying the frequency of adverse animal welfare events such as non-fatal wounding [6]. Without an observer aboard the shooting helicopter it was also infeasible to record the numerical range of bullets fired for each animal. Shooters were aware of what bullet type they were using, and bullet type was swapped after each flight run (approximately two hours). Shooters were asked to assess their level of satisfaction with the efficacy of the bullets used immediately after each flight on a subjective scale of 1–5, as per [43]. The subjective comparison score of satisfaction with performance was framed as speed of incapacitation of shot pigs compared to previously used bullets, and was scored on a scale of one to five, where 1 was much faster than usual, 2 was faster than usual, 3 was no difference, 4 was slower than usual, and 5 was much slower than usual. This was not a blind test as shooters were aware of the bullet type they were using and their judgements may have been affected by their pre-existing attitudes.

Our post-mortem sampling strategy and observation procedures were similar to those described in [11]: we used 4-wheel drive vehicles to locate killed pigs once the shooting helicopter had cleared a geographical zone of the program area. Some killed pigs were also slung underneath the shooting helicopter [44] back to a central location where post-mortems were performed. Based on a sample size simulator for animal-based studies [45], we aimed to perform post-mortem investigations on ~ 200 wild pigs, with approximately half shot with each bullet type. Carcasses of shot pigs not subject to post-mortem examination were left in the environment *in situ* as per standard practice during aerial shooting [14].

Post-mortem examinations were performed on freshly shot carcasses to minimize artefactual post-mortem change. The observer performing post-mortem examinations (ALP) was a veterinarian experienced in collecting data from wildlife shooting programs. We conducted a post-mortem categorization of the location of bullet wound tracts (cranium, neck, thorax, abdomen, limb) following methods used in other published aerial shooting studies [7, 8]. Determining the presence or absence of exit wounds was found to be infeasible for animals that were shot multiple times. Body mass was estimated for each wild pig from morphometric measurements (body length and girth) using an equation published for wild pigs in the US [46].

We then used radiography to assess the terminal ballistic performance of bullets in animal tissues. Radiography is an accepted method in wildlife studies for detecting lead particles [3, 6, 32, 47, 48]. The pigs were radiographed using a portable Cuattro^®^ Slate Plus portable digital radiography system (Cuattro, Golden, Colorado, USA) set at 80 kVp and 2.5 mAs and a wireless 14” x 17” (36 x 43 cm) digital plate [6]. Standardized lateral radiographs were taken of the left side of each pig with the film centered on the middle of the thorax. The radiographs were interpreted by a European board-certified veterinary radiologist (SKJ), who was blinded as to the bullet type used in each film. The presence or absence of metallic fragments or whole projectiles was recorded in radiographs using standard methods [6, 47, 49]. Number of fragments was manually counted to the nearest 10, with a maximum count of 300, i.e. any radiograph with >300 fragments was scored as ‘>300’. The maximum distance (mm) between fragments was also counted for each radiograph as per [48]. Average fragment size for each film was assigned to one of three categories: small (< 1mm), medium (1–5 mm) and large (> 5mm), as per [48]. We estimated average fragment size to the nearest millimeter and the straight‐line distance as the widest point between two fragments in the field of view.

### Statistical analysis

Muzzle kinetic energy was calculated from bullet mass and estimated muzzle velocity for each shot fired during the ballistics trial. Mean muzzle velocity and kinetic energy for the two bullet types were compared using linear models implemented in JAGS [50] called via the runjags package (v2.04–2) [51] in the R statistical environment [52] using 10,000 MCMC draws from each of three chains after discarding 5,000 burn in draws. No statistical tests were applied to data derived from cadaver trials.

For the aerial shooting trial, the mean number of shots fired per wild pig killed was calculated after each shooting run and differences between bullet types were compared using a linear model implemented in JAGS, as described above. The cost per pig killed was calculated within the model as the product of the number of shots and the cost of each bullet type. From post-mortem examinations, mean body mass of pigs shot with each bullet type was estimated using a linear model, as above, and the mean number of bullet wound tracts was estimated using a linear model with Poisson distribution and log link function. From radiography, the number of bullet fragments detected in each pig were counted manually and rounded to the nearest 10, with a maximum count value of 300. The median number of fragments produced by each bullet type was estimated using a linear model with Poisson distribution and log link function. The mean width of bullet fragment fields for each bullet type was estimated using a linear model. The percentage of pigs receiving shots in each of the anatomical zones (head, neck, thorax, limbs, abdomen) and the shot accuracy (percentage of shots hitting a pig) were analyzed using binomial linear models with logit link function. Estimates are reported as back-transformed posterior means or medians, depending on the skew of the posterior distribution, and 95% credible intervals (CrI).

The research was approved by Murdoch University Animal Ethics Committee (O3103/19). The individuals shown in figures in this manuscript have given written informed consent (as outlined in PLOS consent forms) to publish these case details.

## Results

### Ballistic testing

The details of the bullets and their performance are shown in Table 1. Group sizes for the lead-based and lead-free bullets were similar, both < 40 mm. The mean muzzle velocity of the lead-based bullet (890.0 m/s, 95% CrI = 889.4, 890.6 m/s) was 57.5 m/s (95% CrI = 56.6, 58.4 m/s) slower than that of the lead-free bullet (947.5 m/s, 95% CrI = 946.9, 948.1 m/s). Despite their greater mass, the mean muzzle kinetic energy [37] of the lead-based bullet (3337.2 J, 95% CrI = 3336.6, 3337.8) was also slightly lower (8.5 J, 95% CrI = 7.6, 9.3) than that of the lead-free bullets (3345.6 J, 95% CrI = 3345.0, 3346.3). Based on these results, it was considered that both bullet types achieved desirable precision and kinetic energy levels (acknowledging that excessive pass-through energy is undesirable) [6], and were deemed to be appropriate for live animal trials.

**Table 1:** Ballistic metrics for lead-based and lead-free bullets from shooting at paper targets at 50 m. Parameters quantified comprised group size (mm), muzzle velocity, and kinetic energy.

Bullet	Construction	Design	Weight (gr)	Mean group size (mm)	Mean muzzle velocity (m/s)	Mean E_K_ (J)
Speer Varmint^®^	Lead	Hollow-point	130	<40	890	3337
Lehigh Controlled Chaos^®^	Copper	Hollow-point	115	<40	948	3346

### Aerial shooting

We shot a total of 859 pigs over five days, 430 with lead-based bullets and 429 with lead-free bullets. Of these, post-mortem examinations were performed on 100 and 108 pigs respectively. Of these, radiography data of diagnostic quality was derived from 94 and 101 pigs respectively.

For nine shooting runs, lead-based bullets were used exclusively, and for ten shooting runs, lead-free bullets were used exclusively. An average of 0.18 (95% CrI = -0.71, 1.08) more shots were fired per pig during runs in which lead-based bullets were used (4.09 shots / pig, 95% CrI = 3.44, 4.75) than during those in which lead-free bullets were used (95% 3.91 shots / pig, CrI = 3.29, 4.54). However, the probability that the difference between bullet types was > 0 was only 65%. The mean number of bullet wound tracts detected via post-mortem was slightly greater for pigs shot with lead-based bullets (3.29 tracts / pig, 95% CrI = 2.95, 3.65) than for pigs shot with lead-free bullets (2.98 tracts / pig, 95% CrI = 2.66, 3.31) (mean difference = 0.31 tracts / pig, 95% CrI = -0.17, 0.80). There was a 90% probability that the number of wound tracts per pig was greater for lead-based bullets than for lead-free bullets. Based on these figures, 80% of lead-based bullets and 76% of lead-free bullets struck the intended target. Shooters reported identical satisfaction scores (3 out of 5) for 100% of shooting runs, regardless of bullet type used.

Mean body mass of shot pigs was almost identical for those shot with lead-based bullets (70.36 kg, 95% CrI = 70.16, 70.55) and lead-free bullets (70.58 kg, 95% CrI = 70.39, 70.76) (mean difference = 0.22 kg, 95% CrI = -0.05, 0.49). The median probabilities of bullets striking the thorax, abdomen, cranium and limbs were similar for both bullet types, but the odds of a pig being struck in the neck were 2.67 times greater (95% CrI = 1.46, 5.19) (Table 2), during shooting runs in which lead-free bullets were used.

**Table 2:** Probabilities of feral pigs being shot in different body parts with lead-based and lead-free bullets.

Body part	Bullet type	Median	2.5% CrI	97.5% CrI
Thorax	Lead-based	0.60	0.55	0.65
	Lead-free	0.57	0.52	0.62
	Difference	0.03	-0.05	0.11
Abdomen	Lead-based	0.24	0.20	0.29
	Lead-free	0.24	0.19	0.29
	Difference	0.00	-0.06	0.07
Cranium	Lead-based	0.08	0.05	0.11
	Lead-free	0.06	0.04	0.09
	Difference	0.02	-0.02	0.06
Neck	Lead-based	0.04	0.03	0.07
	Lead-free	0.11	0.08	0.15
	Difference	-0.07	-0.11	-0.03
Limb	Lead-based	0.03	0.01	0.05
	Lead-free	0.02	0.01	0.04
	Difference	0.01	-0.01	0.04

Estimates are posterior medians and 95% credible intervals.

**Fig 2:**
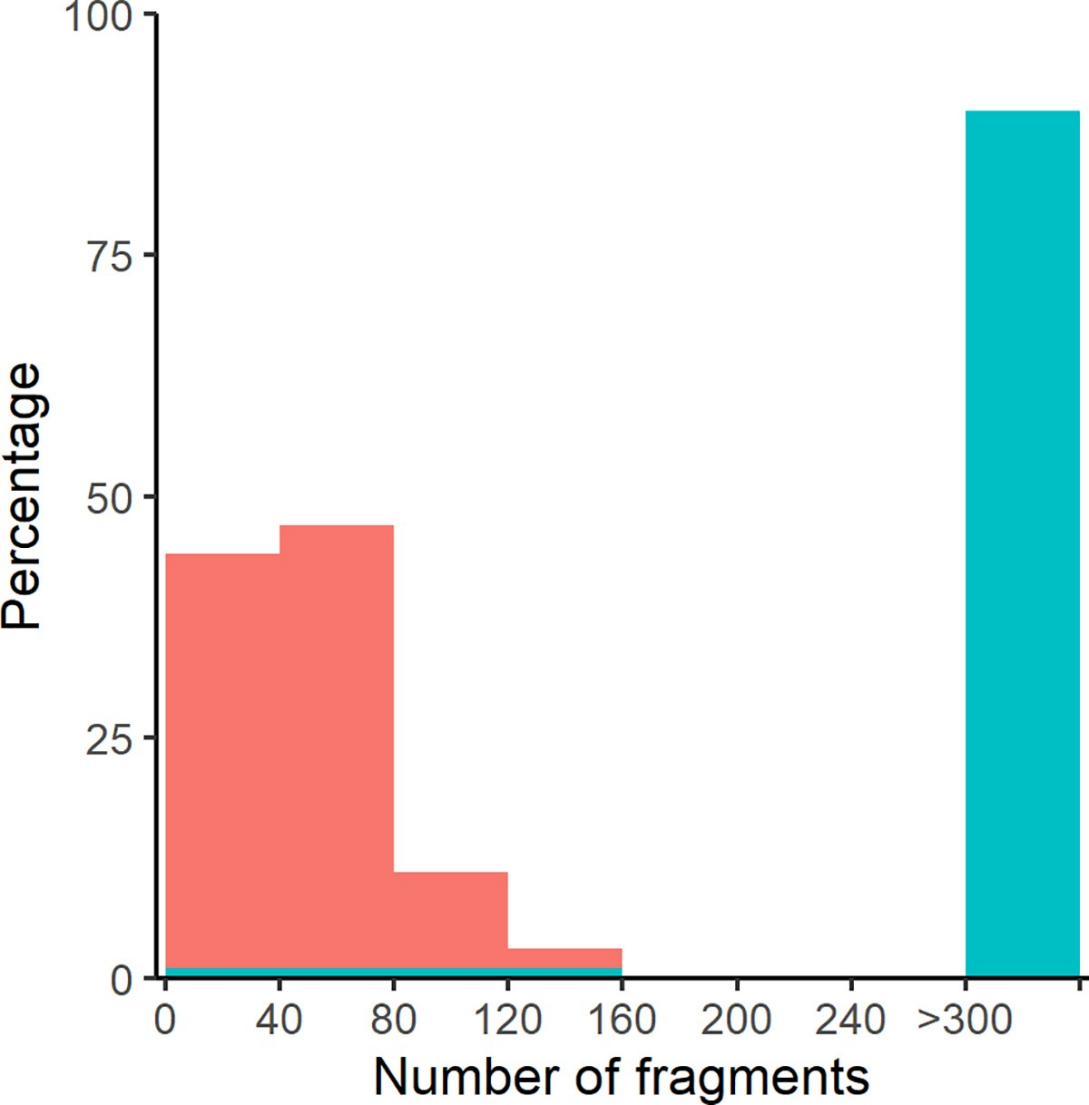
Histogram of bullet fragment number counted in radiographs of wild pigs shot with lead-based and lead-free bullets. The histogram shows that 96% of wild pigs shot from a helicopter with 130 gr lead-based bullets (blue) had >300 visible fragments while 88% of those shot with 115 gr lead-free bullets (red) had <100 visible fragments.

Radiography results indicated that metallic fragments were visible in all pigs. For 95.7% of pigs shot with lead-based bullets, >300 visible bullet fragments were visible, while 89.1% of pigs shot with lead-free bullets had <100 visible fragments (Fig 2). Radiography revealed that the median number of bullet fragments was > 5.3 times greater (95% CrI = 5.2, 5.5) in pigs shot with lead-based bullets (median number of fragments = >300, 95% CrI = 287.5, >300) than in pigs shot with lead-free bullets (median number of fragments = 54.6, 95% CrI = 53.2, 56.1). However, exact numbers of fragments were not discernable as fragment fields often extended beyond the edge of each film, a product of large animal size and relatively small plate size.

**Fig 3:**
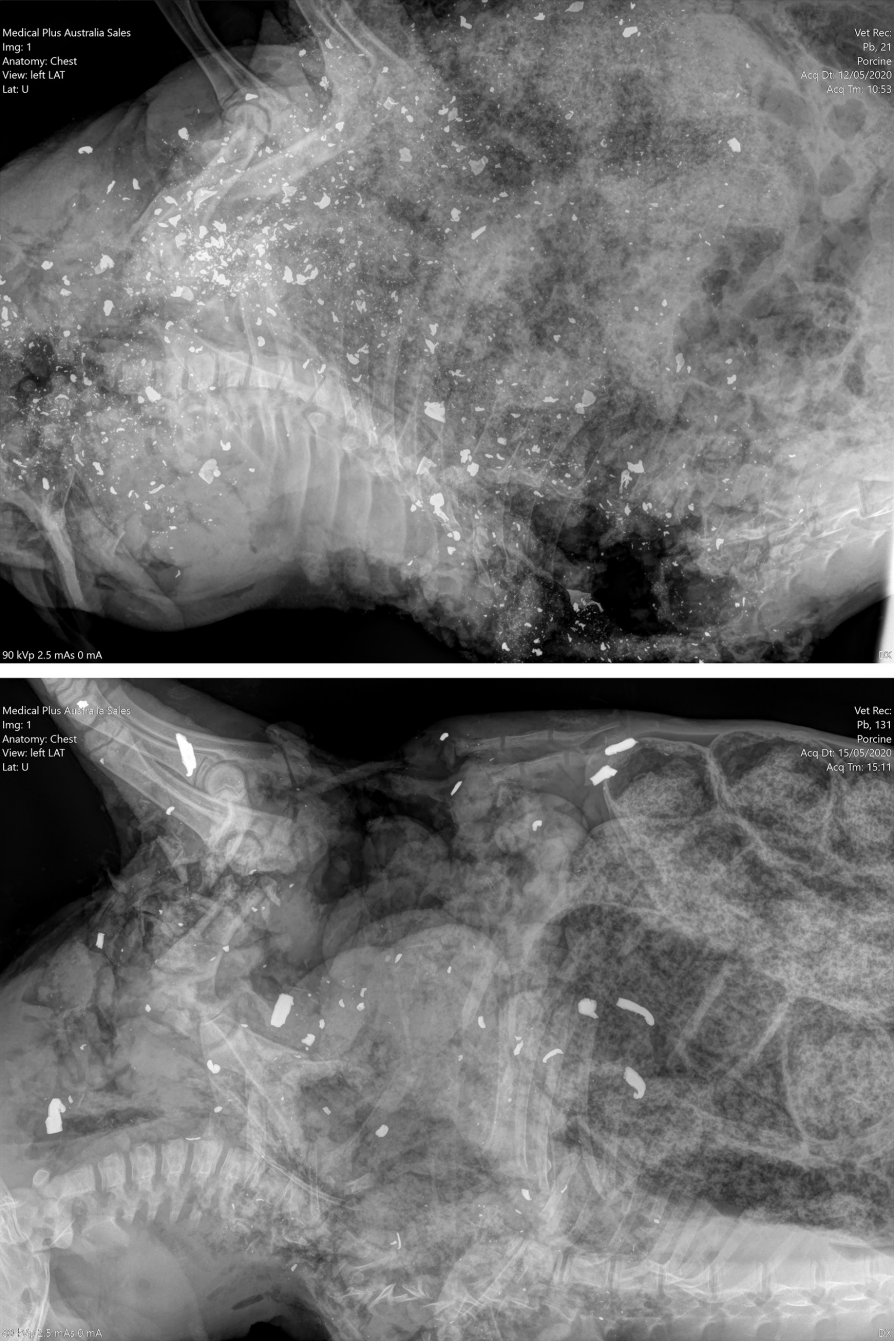
Orthogonal digital thoracic radiographs of wild pigs shot with lead-based and lead-free bullets. Orthogonal digital thoracic radiographs of wild pigs shot from a helicopter with 130 gr lead-based bullets (A), and 115 gr lead-free bullets (B). The thorax was the intended point of impact. There are > 300, typically small, (<1 mm) visible metallic fragments in the wild pig shot with lead-based bullets, (A) and < 50, typically larger, (>5 mm) visible metallic fragments in the wild pig shot with lead-free bullets (B).

Fragment size varied, with average fragment size being classed as small (< 1 mm) (Fig 3A) for 100% of lead-based bullet films and large (>5 mm) (Fig 3B) for 100% of lead-free bullet films. The typical pattern of hundreds of tiny metallic fragments produced by frangible lead-based bullets has been described as a “lead snowstorm” [49] and was consistently observed in the present study (Fig 3A). The mean width of fragment fields was 3.9 cm greater (95% CrI = 3.7, 4.2) for lead-based bullets (28.9 cm, 95% CrI = 28.7, 29.1) than for lead-free bullets (25.0 cm, 95% CrI = 24.8, 25.2).

The costs of purchasing lead-based and lead-free ammunition was AUD$1.31 and AUD$1.80 per cartridge, respectively. The mean cost per pig killed wild pig was AUD$5.36 for lead-based bullets (95% CrI = $4.51, $6.23) and AUD$7.04 for lead-free bullets (95% CrI = $5.93, $8.15), a difference of AUD$1.68 (95% CrI = $0.29, $3.10) per pig.

## Discussion

Our results demonstrated that fragmenting lead-free bullets produced comparable efficacy to lead-based bullets for the aerial shooting of wild pigs. To our knowledge, this is the first study to report the use of lead-free bullets for aerial shooting. Accuracy and precision were similar for both bullet types, as was muzzle kinetic energy, despite the difference in bullet weight (130 gr vs. 115 gr), owing to the higher muzzle velocity of the lead-free bullets. Costs were comparable with lead-free cartridges ~27% more expensive than lead-based cartridges, but this difference ($1.47 per cartridge), while considerable, is relatively minor in the context of operating costs for an aerial shooting program [53]. Radiographs revealed that wild pigs shot with lead-based bullets represent a considerable threat to scavenging wildlife due to the large quantity of lead widely distributed in small fragments in each carcass. The greater cost of the lead-free bullets could be weighed against the environmental benefits of avoiding lead contamination of shot carcasses and the surrounding environment.

Several recent studies have shown comparable outcomes for lead-based and lead-free centerfire ammunition for ground-based shooting of large mammal species. These studies have evaluated shooting of large ungulates such as roe deer (*Capreolus capreolus*) in the United Kingdom [43] and Scandinavia [54], elk (*Cervus elaphus*/*canadensis*) in the US [36], and moose (*Alces alces*) in Scandinavia [35]. At least two published studies have assessed wild pigs. One published study assessed lead-based and lead-free bullets for recreational hunting of wild pigs in Germany and reported comparable results [24]. The aims of these studies and the methods used to quantify their outcomes have varied considerably [6]. While some studies have attempted to compare animal welfare outcomes [55], others have focused on food safety risks [34]. Some studies have used tissue simulants such as ballistic gel or soap [56] without progressing to live animal trials, a useful approach for newly developed firearms technology before undertaking live animal trials [57, 58].

Our results differ from other studies assessing lead-free ammunition in several ways. First, we assessed aerial, rather than ground-based shooting, necessitating quantification of different efficacy metrics. Second, we assessed a single type of lead-free bullet, rather than a range of commercial options. Third, we assessed fragmenting, rather than monolithic copper bullets (e.g. [34]). Fourth, we were unable to quantify anything approximating duration of suffering for shot animals: time to death [6], distance to incapacitation [55] etc. Logical additions to the present study would have been cadaver tests to examine bullet penetration and fragmentation [48], prior to live animal trials, and testing flight distances in wild pigs shot via ground-based shooting as per several recent studies [4, 54, 55]. We were unable to perform these steps but invite future studies to quantify these metrics. The present study had several limitations, including shooting at a single species, using single types of lead-based and lead-free ammunition, and being unable to collect ante-mortem data to quantify speed of incapacitation. Reflection on the limitations and weaknesses of this study lead some of the co-authors to develop a formal multi-stage approach to testing new ballistic technology [58].

We do not suggest that the results of the present study are indicative of lead-free performance for aerial shooting of all species. Further studies are required to assess the efficacy of lead-free bullets for aerial shooting of larger species (e.g. sambar deer (*Rusa unicolor*) [59]) and the appropriateness of fragmenting bullets for such species with thicker anatomical structures and heavier bones. The lead-free bullets used in the present study would likely be suitable for live animal trials of similar sized (30–100 kg) wildlife species commonly targeted in aerial shooting, such as feral goats (*Capra hircus*) and fallow deer (*Dama dama*) [60].

The main arguments for the use of lead-free ammunition are to prevent harm to scavenging wildlife through lead exposure, to human consumers of game meat [61] and to prevent lead accumulation in the environment [62]. Very few animals killed via aerial shooting are used for human consumption, effectively negating risks to humans. Risks of environmental contamination with lead from aerial shooting are apparent. With accuracy rates ~80% for striking wild pigs from a helicopter, an average of ~0.8 bullets per animal killed missed the target and were deposited in the environment. In addition, the remainder of each lead-based bullet that does not fragment stays in the animal (not available to scavengers), but will also eventually be deposited in the local environment once the body decomposes. However, the risk that these bullets pose to harming individual animals appear to be minimal. These effects aside, risks to scavenging wildlife from fragments in carcasses are likely to be of greatest concern for aerial shooting [14].

We argue that aerial shooting of smaller ungulate species (< 150 kg) is likely to pose the greatest threat of harmful lead exposure to scavenging wildlife of all shooting methods for several reasons. First, frangible (‘varmint’) but relatively heavy (~130 gr) lead-core (not bonded) bullets are used. Bonded lead-core bullets tend to be used for larger ungulates and lose less of their mass to fragmentation when compared to lead-core bullets, e.g. 10–24% and 18–27% respectively for moose (*Alces alces*) hunting [35]. Second, animals are shot multiple times due to the deliberate overkill policy [8] (~3 bullets per animal in the present study), compared to ~1 bullet per animal for professional harvesting [63], and 1–2 bullets per animal for recreational hunting [35]. Third, shot animals are left to lie (‘culling-to-waste’) with no removal or meat or organs by shooters; hence all bullet fragments are available to scavengers [14].

Lead fragments from aerial shooting programs in Australia pose risks to raptors such as wedge-tailed eagles (*Aquila audax*) [64–66]. If the bullet weight loss percentages calculated from Scandinavian moose (24% for lead-core 0.308-calibre bullets) [35] is taken as a minimum estimate of fragmentation, the average amount of lead available to scavengers as small fragments would be approximately 7 g per wild pig carcass, or at least three toxic doses for a wedge-tailed eagle [14]. For an average of ~20,000 wild pigs killed via aerial shooting each year in NSW alone (G. Eccles, unpublished data), the total number of eagle toxic doses would be approximately 67,000. This is a minimum estimate of the number of animals potentially affected for two reasons. First, although we did not measure bullet weight loss in this study, the radiographic evidence of “lead snowstorms” [49] suggest that a higher proportion of bullet weight loss occurs in this type of ‘varmint’ bullet design than in lead-core bullets used for moose hunting. Second, if less conservative interpretations were used for lethal doses of lead in eagles, or smaller raptor species were considered [35], estimates of the number of lethal avian doses produced would be much higher [14].

There is also the possibility that the large, sharp and hard (compared to lead) copper fragments produced by the lead-free bullets could cause mechanical (not chemical) damage to the gastro-intestinal tracts of scavenging animals. While the potential for copper toxicity has been assessed for lead-free bullets [34], we are unaware of any studies to assess the potential for mechanical trauma from large copper bullet fragments.

Our results suggest that lead-based and lead-free bullets are similarly effective for aerial shooting of wild pigs, but that the bullet types behave differently, with lead-based bullets displaying a higher degree of fragmentation. Aerial shooting may be a particularly important contributor to scavenging wildlife being exposed to lead and investigation of lead-free bullets for this use should continue. Our results emphasize the importance of considering aerial shooting in discussions of transitioning to lead-free bullets.

## Supporting information

S1 Data (CSV)

S2 Data (CSV)

S3 Data (CSV)

S4 Data (CSV)
